# *From existential uncertainty to a new mindset promoting recovery*: Exploring the development of uncertainty experience in women with vulvar neoplasia – A qualitative longitudinal study

**DOI:** 10.1186/s12905-024-02889-4

**Published:** 2024-01-13

**Authors:** Jasmin Eppel-Meichlinger, Hanna Mayer, Enikö Steiner, Andrea Kobleder

**Affiliations:** 1 Department of General Health Studies, Division Nursing Science with focus on Person-centred Care Research, Karl Landsteiner University of Health Sciences, Dr. Karl-Dorrek-Straße 30, Krems, Austria; 2 Vienna Doctoral School of Social Sciences, University of Vienna, Vienna, Austria; 3 Department of Obstetrics and Gynecology, Vienna General University Hospital, Vienna, Austria; 4 Institute of Applied Nursing Science, Eastern Switzerland University of Applied Sciences, St.Gallen, Switzerland

**Keywords:** Vulvar Neoplasms, Uncertainty in illness, Oncology nursing, Qualitative research

## Abstract

**Background:**

Women with vulvar neoplasia continue to experience uncertainty up to six months post-surgery. Uncertainty in illness is considered a significant psychosocial stressor, that negatively influences symptom distress, self-management strategies and quality of life. According to the *Reconceptualized Uncertainty in Illness Theory*, the appraisal of uncertainty changes positively over time in chronic illness. We aimed at exploring whether and how the experience of uncertainty develops in women with vulvar neoplasia.

**Methods:**

We selected a purposive sample of seven women diagnosed with vulvar neoplasia in four Swiss and one Austrian women’s clinic. By means of a qualitative longitudinal study, we conducted 30 individual interviews at five points of time during one year after diagnosis. We applied Saldaña’s analytical questions for longitudinal qualitative research.

**Results:**

First, participants experienced uncertainty as an existential threat, then an inherent part of their illness, and finally a certainty. Women initially associated the existential threat with a high risk for suffering from severe health deteriorations. Participants that could reduce their individually assessed risk by adopting health promoting behaviors, accepted the remaining uncertainty. From now on they reframed uncertainty into a certainty. This new mindset was based on a belief of promoting recovery and reducing the risk of recurrence.

**Conclusions:**

The long-lasting and oscillating nature of uncertainty should receive attention in supportive oncology care. Uncertainty concerning existential issues is of special importance since it can inhibit a positive development of uncertainty experience.

## Background

Uncertainty is a significant phenomenon in the illness experience of persons with an oncological disease during their illness trajectory [1]. It is not only limited to the phase of diagnosis and treatment, the experience of uncertainty can persist when oncological therapy has already finished, and affected persons have reached the phase of survivorship. Diagnosis, treatment, medical follow-ups as well as personal and social issues such as work, relationships, and identity are associated with the experience of uncertainty [2, 3]. It can negatively affect physical, psychological, and existential outcomes [4]. Greater uncertainty is associated with increased fatigue, insomnia [5], emotional distress [4], anxiety, depression [6] and lower quality of life [7] and can also influence psychosocial adjustment to the diagnosis of cancer [8].

A variety of studies have investigated uncertainty in individuals with several types of cancer at various stages of the disease trajectory [9–11]. In a longitudinal study, Raphaelis, Mayer [12] showed that uncertainty was highly prevalent in women with vulvar neoplasia throughout the course of six months after diagnosis. Vulvar neoplasia includes vulvar cancer and vulvar intraepithelial neoplasia (VIN) as precancerous cellular change in the external female genitalia [13]. Although vulvar neoplasia is a rare disease, its incidence has increased globally over the past decade, especially in younger women [14]. Mostly, surgery is the first choice of treatment, since there is a limited role for primary radio or chemotherapy [13]. Across all stages, treatment for vulvar neoplasia is associated with significant morbidity and impact on quality of life. Symptoms commonly reported after treatment include bleeding, pain, odour, pruritis, sexual dysfunction, urinary incontinence, constipation, and lower extremity oedema [15]. Women have also reported diminished emotional and social functioning, as well as compromised body image and sexuality, emotional and interpersonal distress, particularly if it requires extensive resections of the labia or clitoris [12, 16–18]. According to Senn, Eicher [17], uncertainty is one of the most prevalent psychosocial symptoms, occurring in about 83% of women with vulvar neoplasia [17]. Their experience of uncertainty refers to the risk for disease transmission, progression, and recurrence. Affected women reported about uncertainty with regard to their reproductive and sexual capacities after treatment completion. In addition, vulvar neoplasia remains a stigmatised condition associated with poor hygiene or promiscuity [16]. Affected women felt isolated and ashamed to speak about their condition and their experienced uncertainty [19, 20]. This tendency to not talk about the disease because of its location and societal associations may reinforce illness-related uncertainty [19, 21, 22].

The phenomenon of uncertainty in illness was theoretically framed by the work of Mishel [23–25]. She first developed the Uncertainty in Illness Theory (UIT) [25] and defined uncertainty in illness as the inability to structure the meaning of illness-related events cognitively because of insufficient information. The Reconceptualized Uncertainty in Illness Theory (RUIT) was developed two years later with awareness of the limitations of the UIT, where the development of uncertainty was viewed linearly [26]. The theory was reconceptualized through discussions with colleagues and qualitative data from patients with chronic conditions. Finally, RUIT addresses the experience of continuous uncertainty, such as in a chronic or potentially recurring illness. It is the central theoretical proposition of RUIT that the appraisal of uncertainty in chronic illness changes over time – from a danger to an opportunity [24]. As result of this process Mishel described growth toward a new value system, whereas the result of the UIT is a return to the previous level of adaptation [24]. Predominantly qualitative studies have provided empirical support for the RUIT. They affirmed a transformative process that is characterized by the transition towards a new orientation where uncertainty is accepted as an inherent aspect of life [27]. This process was described in various ways by researchers, including themes such as “developing a revised life perspective”, “finding new ways to navigate the world”, “experiencing growth through uncertainty”, “achieving new levels of self-organization”, “setting new goals for living”, “devaluing previously important things”, “redefining what is considered normal”, and “creating new dreams” [28]. In all studies the gradual embrace of uncertainty and the restructuring of one’s own reality were identified as significant phenomena of the process, aligning with the assumptions of the RUIT. However, the support of the RUIT differs by population and methodology, i.e., more qualitative than quantitative studies confirmed the RUIT. The samples of these studies included breast cancer survivors [29], women regenerating after cardiac disease [30], chronically ill men [31], HIV patients [32], long-term diabetic patients [33], persons with schizophrenia [34], women who have not been diagnosed but were genetically predisposed to hereditary breast and ovarian cancer [35], spouses of heart transplant patients [36], and adolescent survivors of childhood cancer [37]. Although several empirical works supported the proposition of the RUIT, the results have not yet been fed back to the theory in a synthesized form. As a result, it is still not clear in an explanatory manner *how* uncertainty develops in the chronic course of a disease from a danger to an opportunity.

Nevertheless, Mishel’s theoretical considerations opened a new perspective on the phenomenon of uncertainty in chronic illness, such as in cancer, and potential opportunities for the discipline of nursing to intervene therapeutically in the illness trajectory. This is especially relevant for women with vulvar neoplasia as a group characterized by a high recurrence rate [38] and by taboo-related communicative difficulties [20]. While it is already known that women with vulvar neoplasia experience uncertainty up to six months after diagnosis [12], it is unclear whether and how their experience of uncertainty changes during the chronic illness trajectory and how the findings can inform the further development of the RUIT.

## Methods

We aimed to explore the development of uncertainty experience in women with vulvar neoplasia over time and to discuss the significance of the results for Mishel’s RUIT [24].

### Design

We conducted a longitudinal qualitative study since we intended to inductively explore the unexplored development of uncertainty over time. For the purposive sample, we included women aged 18 years and older with vulvar neoplasia (initial diagnosis or recurrence) who were about to receive surgical treatment. Between May 2019 and January 2021, gynaecologic oncology nurses invited women of four Swiss and one Austrian women’s clinics to participate in this study.

### Data collection

Data collection took place via qualitative interviews, depending on the participants´ preferences, face-to-face in the hospital or at home, via phone or video call. We recorded the interviews digitally. In addition, notes were taken during and after each interview, which were included in the analysis. Participants were invited to bring a trusted person to the interview. None of the women made use of this option. The first author, a female PhD candidate having a nursing background and working as research associate at a University of health sciences, conducted the interviews at the following points of time: (1) at diagnosis or before surgical treatment, (2) one week later, (3) six months later, (4) nine months later and (5) one year later. The interviewer and the participants met for the first time at the time of the 1st interview. The first three points of time were chosen for reasons of explanatory power according to the results of Raphaelis et al. [12]. We chose the other points of time with an exploratory intent.

We developed a semi-structured interview guide consisting of four central subjects including both backward and forward looking questions to explore processes and change over time [39]. We adjusted it after the first three interviews with participants regarding the degree of abstraction of the narrative stimuli. The central topics were: (1) Current status related to the vulvar neoplasia, (2) situations of uncertainty, (3) retrospective reflections on developments over time, (4) outlook for further therapy or the recovery phase.

### Data analysis

We first conducted within-case analyses for the trajectory of each participant. Afterwards, we performed a cross-case analysis for reasons of comparison, thereby intending to reach a higher level of abstraction and to develop a theoretical model. For data management and analysis, we used MAXQDA22© software [40].

To explore the individual temporal trajectories of the participants, each individual interview was analyzed separately by the first author. Since we were interested in changes of participants’ uncertainty experience also on a theoretical level, the coding strategy of Grounded Theory was followed [41]. In a first step, the data were openly coded, followed by axial coding in order to develop initial concepts. To systematically identify changes over time for each participant, we conducted a longitudinal analysis using Saldaña’s [42] framework for longitudinal qualitative research. Framing, descriptive, analytic, and interpretative questions guided the identification of changes over time.

To identify similarities and differences, we performed cross-case analyses. By means of a second coding cycle, the single cases were merged into generic sub-categories and broader categories in order to compare them. Thereby axial and selective coding was used [41]. Finally, we synthesized these results in a central model in order to explain the common development of uncertainty experience over time.

### Trustworthiness

To enhance the trustworthiness of our findings, we adhered to the criteria of adequacy, empirical saturation, and theoretical pervasiveness for qualitative social research [43]. Therefore, we identified the research question from the field of interest and established it against the background of the theoretical work by Mishel [24] (adequacy and theoretical pervasiveness). We empirically collected data and analyzed them inductively (empirical saturation).

### Ethics

Participation was voluntary and could be withdrawn at any time without giving reasons. Informed consent was ongoing processed at each interview. In addition to study information, the researcher’s role as a PhD student in the study was disclosed.

## Results

We conducted 30 interviews between November 2019 and November 2021. Each of the seven participants completed three to five interviews. Four of seven participants completed all five interviews. One participant passed away during the study period due to a postsurgical bleeding, one participant could no longer be reached by telephone after the third interview and another after the fourth. The length of the interviews ranged from 13 to 75 min (Mean = 40).

### Characteristics of the participants

Five women from Austria and two from Switzerland participated in our study. Their age ranged between 28 and 85 years. Four participants were diagnosed with vulvar cancer, three with vulvar intraepithelial neoplasia. Four had an initial diagnosis (Table 1).

**Table 1:** Characteristics of the participants

# Participant	Nationality	Age in years	Diagnosis	Pre-existing chronic condition	Disease-related events during the study period
1	Switzerland	56	VIN (Initial)	Chronic pain due to nerve injury	Occurrence and prolonged persistence of an erythema
2	Switzerland	64	VC (Initial)	Alcoholism	Delayed wound healing, recurrence
3	Austria	28	VC (Initial)	None	Hospitalization due to postoperative sepsis
4	Austria	67	VC (Recurrence)	None	Persistent pain
5	Austria	80	VIN (Reccurence)	Uterine & cervical cancer	Bladder symptoms and a recurrence
6	Austria	85	VC (Recurrence)	None	None
7	Austria	56	VIN (Initial)	None	None

Abbreviations: VIN = Vulvar intraepithelial Neoplasia, VC = Vulvar Cancer

### Development of uncertainty experience in women with vulvar neoplasia

The experience of uncertainty developed in three stages within one year: (1) uncertainty as an existential threat, (2) uncertainty as an inherent part of illness, and (3) uncertainty as a certainty.

**Fig. 1:**
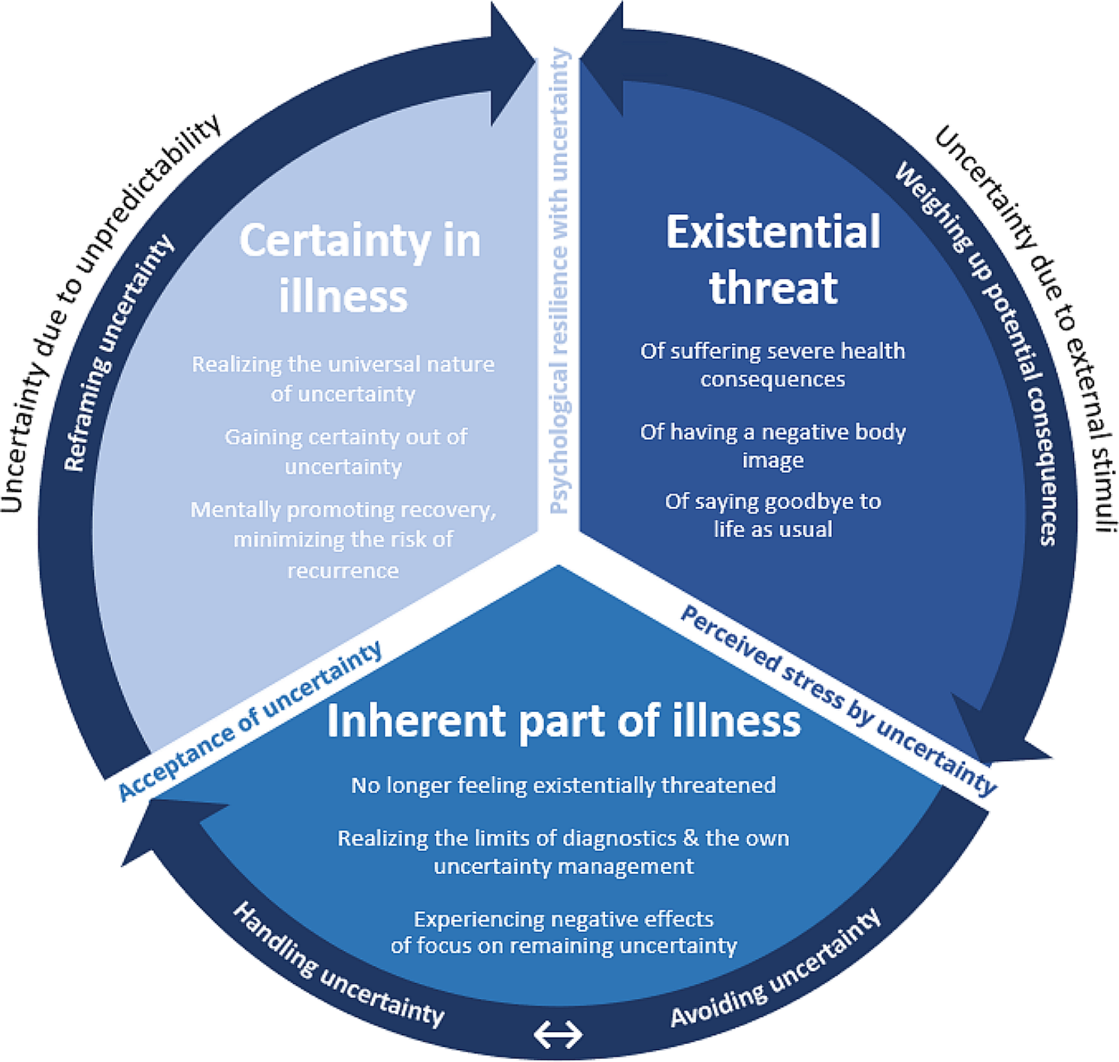
Development of uncertainty experience in women with vulvar neoplasia

The analysis revealed that the experience of uncertainty continuously developed back and forth during the study period of one year. Participants developed different coping strategies in dealing with uncertainty: weighing up potential consequences, avoiding or handling uncertainty, and reframing uncertainty. This fluctuating development of the uncertainty experience is visualized as a cyclical model (Fig. 1).

### Uncertainty as an existential threat: The Sword of Damocles

The unknown meaning of a new symptom, having to wait for the result of an examination, not understanding the meaning of the diagnosis and its consequences, and realizing an increased risk of developing vulvar cancer or a recurrence were stimuli for uncertainty. Participants reacted to the unknown meanings of these uncertainties by creating an explanation based on their existing knowledge or previous experiences, e.g., through a pre-existing chronic condition or a previous vulvar neoplasia. Against this background they implicitly made a judgment of their individual risk for experiencing existential consequences. Participants [3–7] assessing a high risk of potential consequences by the experienced uncertainty felt threatened by the possibility of suffering from serious health deteriorations or dying:

*“On this ward where everyone was practically running around from dying of cancer. That was simply a catastrophe for me. I generally don’t want anyone to end up like that, but I certainly don’t want to”* (Interview 1, Participant 3).

Symptoms triggered existential uncertainty throughout the different health- and recovery phases. They played a significant role in the diagnostic process, when participants first noticed a changed appearance of the vulva. Symptoms continued to re-stimulate existential uncertainty after cancer treatment was completed and their experience of uncertainty had already developed positively. In this phase all the participants again judged their risk for existential consequences and perceived uncertainty a threat due to the possibility of having a recurrence:

*“I was alarmed […] and had a very bad feeling. It could have been that it has come back, but inside which means that it has spread to the lymph nodes”* (Interview 3, Participant 1).

This alarm resulted from the awareness of the increased risk of cancer in participants with a precancerous stage and of recurrence in participants with vulvar cancer. By again weighing up their risk for existential consequences, they once again perceived uncertainty as a threat due to possible cancer recurrence.

Having to wait for the (still) unknown result of an examination occurred several times in the course of the illness trajectory as an uncertainty. This concerned the results of the primary clarification of the diagnosis, and after surgery the histological findings regarding the complete removal of the carcinoma.

*“The worst part was the first two weeks, when I heard from my gynecologist that it was vulvar cancer, but I hadn’t had any further examinations, and it was possible that the cancer had already spread. And I don’t have much longer to live. It still hurts me”* (Interview 2, Participant 3).

Uncertain consequences were the necessity of a further surgery, which would involve, e.g., the removal of the lymph nodes. The consequential possibility of needing an ostomy or a full resection of the vulva because of the uncertain necessity of a radical surgical oncological treatment, threatened participant 2 on the one hand by the risk of experiencing a serious physical change or on the other hand by a “disfigured” intimate area and to not being able to have children in the future (participant 3). Furthermore, the fear that the existing cancer might have metastasized underlay the existential uncertainty (participants 2, 3, 7).

In the longer-term course of the disease or recovery, having to wait for the results of the routine gynecological oncological check-ups was again an uncertainty stimulus for all the participants, even if women experienced no symptoms. Though, available findings were no guarantee to full understanding and comprehension of their meanings to participants. Especially older women (participants 5, 6) experienced uncertainty about the meaning of the diagnosis, and the prospects for their treatment and recovery but would not dare to ask the physician to explain:

*“I think they will try to help me… but to what extent it is possible, that is written in the stars for me”* (Interview 2, Participant 6).

One of the participants associated uncertainty with the phrase of the “Sword of Damocles” (interview 1, participant 7). Living under a “Sword of Damocles” was a recurring experience. At a later stage of disease or recovery, women again had to wait for the results of their routine gynecological oncological check-ups. This recurring experience was always a stimulus triggering uncertainty, even if the participants did not experience symptoms. This uncertainty shaped their experience on an affective level. It was a significant stressor which manifested itself by fear, insecurity, sadness, anger, and the feeling of powerlessness:

*“Well, that’s it – a trembling, an anxiety, like a threat. What will come? But whatever will come, whether good or bad, I have no other choice”* (Interview 1, Participant 6).

### Uncertainty as an inherent part of the illness: An accepted companion

The enduring experience of threatening uncertainty was a starting point for employing coping strategies, either dealing with uncertainty, such as reducing it and mentally processing the negative experience of it, or avoiding uncertainty. Reducing uncertainty involved the acquisition of information to support informed decision-making. However, participants not just reduced uncertainty that was based on a lack of information, but on the (still) uncertain outcome of an investigation or a treatment. Therefore, they adopted health promoting behaviors to minimize the probability of occurrence of the uncertain adverse event, such as having metastases Participants 1, 2, 3 and 7 coped with the threatening uncertainty experience by thinking positively, by practicing self-care, as well as by reflecting about their emotional responses. By thinking positively, they encouraged themselves to hope for the best, to think in a constructive manner and to calm themselves:

*“It depends on how I deal with it. It’s always been like this… I say: What am I suffering from? Nope, I don’t think so. I’m healthy, I’m the greatest, I’m the best, I’m the winner*” (Interview 1, Participant 2).

They found strength in taking uncertainties with a sense of humor and focusing on meaningful things. Their practice of self-care consisted of not letting the stress of the threatening uncertainty get them down, e.g., of letting oneself go (participants 1, 2, 3, 7). Therefore, they promoted their health, not only related to the vulvar neoplasia, by paying attention to their needs, exercising regularly, eating a balanced diet, and reducing other stress factors, e.g., splitting the care of the mother in need of care (participant 1). To cope with the psychological stress of uncertainty, they paid more attention to clearing their minds by engaging in meaningful activities, talking about their fears to people they trust and spending most of their time in familiar surroundings:

*“Getting out into nature… That’s my first priority, that I can somehow manage that and through that I can really switch off”* (Interview 2, Participant 5).

If the interviewees were able to overcome existential uncertainty, e.g., by completing cancer treatment or if a symptom cleared up as harmless, it triggered a change in their experience of uncertainty:

*“When I woke up after surgery and they told me that the lymph nodes were fine, it was such a relief…All was well! All was well! I managed that, I got off lightly”* (Interview 3, Participant 7).

Participants [1–3, 7] experienced uncertainty no longer as an existential threat. Instead, they accepted uncertainty as an inherent part of illness and opened up to the concept of it. They accepted that certainty in illness will probably never exist – despite all the information and expert knowledge of professionals as well as their own coping strategies:

*“I can’t do it myself … determine my own fate, that’s actually presumptuous. You don’t have full control over your own life”* (Interview, Participant 1).

The analysis revealed that the remaining uncertainty did not refer to a specific external stimulus, such as surgery or pending findings anymore. From now on the women’s uncertainty experience mainly concerned the irreducible unpredictability regarding the disease course and the prognosis:

*“It is difficult to estimate the course of an illness. You never know how it will end. It’s just part of the game”* (Interview 1, Participant 2).

Other participants [4–6] who we did not find uncertainty acceptance, reported of repressing uncertainty and its existential threat. This was the case if the interviewees were not able to reduce uncertainty or to cope with it. We found the aim of this avoidance strategy was to restore normality – as if nothing had ever happened. These participants did not want at any price facing uncertainty as a part of their life.

*“I don’t know, maybe I’m a strange person, but I try to repress everything, the senselessness of it all”* (Interview 3, Participant 5).

They kept a mentally distance by distracting themselves, in order to not having to think about uncertainty. Participant 5 rejected new information to avoid getting bad news. They furthermore constructed a negative certainty, i.e., being convinced that the uncertainty will in fact occur:

“At the moment I feel better because now I know that the cancer is there and that it won’t go away” (Interview 5, Participant 5).

Unlike the other participants, they could not accept uncertainty as a result of their management strategies, but rather gave up under the feeling of having no choice and resigned themselves to the uncertainty and the threat that came with it. Participants implementing the avoidance strategy consequently reported feeling depressed and powerless. They had a little sense of control, as they felt they have no choice.

### Uncertainty as a certainty in illness: A mindset to promote recovery

As participants 1, 2, 3 and 7 had accepted uncertainty, they increasingly observed a positive impact on their recovery and health. As a consequence, they developed a new mindset – with uncertainty in the background and their awareness about it in the foreground. They were convinced that an altered cognitive focus made a positive impact on their recovery and would reduce their risk of cancer recurrence. The new mindset regarding uncertainty was characterized by the realization of the universal nature of the phenomenon. They no longer felt alone with uncertainty in their illness as soon as they became aware of the certainty of uncertainty as a natural part of life - that concerns all aspects of human existence:

*“Uncertainty for me means…. It’s not just me, everyone is affected by it, I can only take each day as it comes and then solve the problems”* (Interview 4, Participant 1).

In their new mindset women gained trust in their psychological coping strategies. They concluded that their own perspective made a difference and improved their sense of control. This mindset allowed them to experience increased self-confidence in being able to beat cancer. They felt relieved, reported more serenity, mental closure, mental health, and resilience:

*“I have processed it mentally; I am in a positive mood. I am convinced that this makes a difference. Whether something comes back or not […], because the physical and the psychological are so close together, you can’t separate them”* (Interview 2, Participant 2).

## Discussion

This longitudinal qualitative study explored how the uncertainty experience developed in women with vulvar neoplasia over the course of one year. The findings were not only of phenomenological interest, but also of theoretical as the study was conducted with sensitivity of the Reconceptualized Uncertainty in Illness Theory [24]. They contribute to a deepened understanding of the uncertainty experience of women with vulvar neoplasia in the illness trajectory but also inform the further development of Mishel’s theory itself.

The development of uncertainty was never complete but oscillating in the chronic course of the disease in women with vulvar neoplasia. Change in uncertainty experience was inhibited by existential uncertainty and promoted by the acceptance of uncertainty. According to the RUIT [24], uncertainty experience changes in a positive way when someone is at the peak level of instability due to uncertainty. In this qualitative longitudinal study, we also identified participants experiencing instability due to threatening uncertainty. Over time, however, we did not observe a development in the experience of uncertainty, as long as uncertainty still was perceived existentially threatening. However, we found commonalities regarding the re-appraisal of uncertainty under other circumstances. In a similar vein as Mishel [24], the participants described a new view of life allowing a change of perspectives with regard to evaluation of uncertainty. However, this development did not lead to perceiving uncertainty as an opportunity. In our study, the development of uncertainty occurred in individuals who were able to reduce the threat of uncertainty or where uncertainty dissolved by external circumstances. Subsequently they did not associate the uncertainty with existential consequences but were still experiencing uncertainty that, however, concerned the unpredictability of the further illness trajectory.

In accordance, Han et al. [44] suggest that existential uncertainty may be a bigger threat for patients than more information-related aspects of uncertainty, i.e., uncertainty associated with diagnosis, prognosis, causal explanations, and treatment. Existential uncertainty encompasses an awareness of the fact that one`s own existence is undetermined but finite. Being existentially uncertain means to live with a constant threat to one’s own existence – a threat reaching beyond the physical domain and affecting the social, personal, and spiritual domains. Dwan and Willig [45] outlined key distinctions between existential uncertainty and other aspects of uncertainty in the experience of persons with cancer. Thereby, the focus is on meaning rather than on facts, on the person rather than on the disease, and the fundamental nature of the human being in the world. Another comparable distinction of existential uncertainty was made by Karlsson, Friberg [46], as they characterized it as living with an unpredictable future, being confronted with one’s own impending mortality, and undergoing personal development. Against this background Penrod [47] cautioned that providing even more information to reduce existential uncertainty may be counterproductive to change the negative experience of uncertainty. However, existential uncertainty that could be overcome may result in existential well-being, that similarly depends on a person’s meaning and purpose of life, and feelings regarding death and suffering [48]. A bidirectional relationship of existential well-being and health-promoting behaviours is assumed [49]. This would explain why individuals changed their perspective on uncertainty as a health-promoting behaviour, as soon as uncertainty was no longer experienced as existentially threatening, but could be accepted, perhaps leading to existential well-being. Greater manifestation of existential well-being is associated with a reduced incidence of depression and an improved overall health condition. Existential well-being is furthermore connected with emotional well-being, that manifests as social engagement, health-promoting behaviours, and positive affect and optimism [50]. A positive mindset and proactive living promote the relationship of existential well-being and health-promoting behaviours. In particular, having a positive mindset to foster health was influenced by a positive self-image, and having sense of control [51]. Having a positive mindset and sense of purpose in life were directly associated with health-promoting behaviours of proactive living [52]. Especially in individuals living with chronic illness, looking for and having meaning are positively correlated. Following, the promotion of finding meaning in life should have high priority during the management of chronic disease [53] to overcome existential uncertainty and achieve existential well-being.

### Limitations and strength of the study

A limitation concerns the short duration of some interviews. Extended interviews would contribute to an in-depth understanding of individual experiences and to draw conclusions with regard to a longer period of time. Furthermore, due to the peculiarities of the group of interest of women with vulvar neoplasia, the transferability of the results to persons with another oncological condition or chronic disease is limited. However, the longitudinal study design contributes to uncover dynamic processes as they occur and to offer insights into changes and continuities within the life course [54].

### Implications

The results of this study show that the experience of uncertainty changes over time in women with vulvar neoplasia, since different types of uncertainty occured during the illness trajectory. Uncertainty associated with existential consequences did not develop in a positive way until participants were able to cope with it. It is therefore important to differentiate between several types of uncertainty. The role of existential uncertainty should be considered as potential inhibitor of change in the interaction with women with vulvar neoplasia and with regard to intervention planning. In the context of cancer, there is growing evidence that meaning-oriented uncertainty interventions might be most useful [55–57]. The combination of existential uncertainty and the identified possibility of change in the experience of uncertainty emphasize the need to develop an own language and understanding of professionals in order to anticipate and address different aspects of patients´ uncertainty experience [58].

## Conclusions

The findings provide health care practitioners, especially in the field of psycho-oncology with a deeper understanding of the development of uncertainty experience in the disease trajectory of women with vulvar neoplasia. Our results may inform practice, in particular interactions with affected individuals. Furthermore, the findings strengthen the theoretical basis of uncertainty in chronic illness. They can provide orientation for developing theory-based measurements and interventions. Finally, we reflected the results against the background of the RUIT [24]. Thereby, the results can contribute to theory dynamics in nursing, by informing theory further development and adding to the body of existing theories.

## Data Availability

The datasets used and/or analysed during the current study are available from the corresponding author on reasonable request.
